# Microbiological Safety of Cut Melons Sold in Portuguese Retail Markets: A Pilot Study

**DOI:** 10.3390/foods11244010

**Published:** 2022-12-11

**Authors:** Yu Hsuan Tseng, Joana Barbosa, Teresa Bento de Carvalho, Paula Teixeira

**Affiliations:** Universidade Católica Portuguesa, CBQF—Centro de Biotecnologia e Química Fina—Laboratório Associado, Escola Superior de Biotecnologia, 4169-005 Porto, Portugal

**Keywords:** foodborne pathogen transfer, hazard, microbiological quality, *Escherichia coli*, *Listeria monocytogenes*, *Salmonella* spp.

## Abstract

Due to the increasing consciousness of a healthy diet and pursuit of convenience among consumers, the market for fresh fruit is on the rise, and the melon is among the most welcome of fruits for its sensory attributes and nutritional properties. Consumption safety of cut fruit remains an issue of concern that may affect public health. This study aimed to perform the microbiological characterisation of a melon, *Cucumis melo* L. var. “Piel de Sapo”, cut by retailers, wrapped in plastic cling film and kept at room temperature in local fruit shops. In addition, the possible transfer of relevant foodborne pathogens, during slicing, from the peel to the interior of the melon, and bacterial growth, were also evaluated when the melon slices were stored at abusive temperatures for 2 days. In this pilot study, a low number of samples were characterised microbiologically (26 cut melons), and some isolates were identified by 16S rRNA sequencing. No *Listeria* spp. or *Salmonella* spp. were detected in any of the samples, while *Escherichia coli* and *Staphylococcus aureus* were present in four and six out of twenty-six samples, respectively. Following artificial contamination of melons with cocktails of *Salmonella* spp., *E. coli* and *Listeria monocytogenes*, it was observed that, despite the smaller number of *L. monocytogenes* recovered, all the pathogens were transferred from the contaminated peels to the interior of the melons. Furthermore, over storage time, significant differences were observed (*p* < 0.05) between the counts obtained from melon slices immediately after cutting (0 h), and after 24 and 48 h at 20 °C, with an increase of about 4 log CFU/g in all the pathogens. In conclusion, some cut melons classified as microbiologically unacceptable or unsatisfactory are being sold in local fruit shops in the Porto Metropolitan Area, Portugal. Although absent in the samples analysed, *Salmonella* spp. and *L. monocytogenes*, if present, can be transferred from the outside to the inside of the fruit by the cutting blade and, if not consumed immediately and stored at abusive temperatures, this ready-to-eat product poses a risk of infection. This pilot study, performed for the first time in Portugal under these conditions, clearly demonstrates the need for education campaigns to alert local sellers and consumers of the risk posed by cut melons.

## 1. Introduction

Fresh-cut fruits, including cut melons, have been widely introduced into our diet recently due to their convenience and practicality in daily life. Although melons do not undergo any processing before being consumed, they have emerged as vehicles for foodborne diseases, with transportation chains from producer to the consumer being a highly probable contamination source [1]. Since the melon is part of the *Cucurbitaceae* family, crop growth occurs on the ground, making the fruit susceptible to contamination through contact with pathogens found in soil or irrigation water [2,3]. Fruits, subjected to peeling and/or cutting, are vulnerable to natural processes that will promote the perishability of the products, as well as favour bacterial growth.

The process of cutting easily permits contamination of the fruit flesh: several outbreaks were associated with the consumption of fresh-cut fruits contaminated with pathogens such as *Salmonella* spp., Shiga toxin-producing *Escherichia coli*, *Campylobacter* spp., *Staphylococcus aureus* and *Listeria monocytogenes* [4,5,6,7,8]. In 2019, an outbreak of salmonellosis (*Salmonella* Carrau) in the United States was linked to cut melon products, namely, watermelon, honeydew, cantaloupe and mixed fruit melons. From the 137 reported cases, 38 cases were hospitalised. The outbreak spread to 10 states, with the product being recalled from all stores. The Food and Drug Administration (FDA) conducted epidemiological and trace-back investigations, but the contamination source of the melons was not identified for this outbreak [9]. In 2021, another outbreak of salmonellosis (*Salmonella* Braenderup ST22) was identified, with cases reported by multiple European countries. Galia melons imported from outside the EU/EEA were thought to be the possible vehicle of infection. A total of 348 confirmed cases were identified in 12 countries, with 68 people being hospitalised and no reported deaths. The contamination source was traced to a melon production facility in Honduras [10]. Zhang et al. [11] studied the microbiological safety of fresh produce in Canadian retail markets, finding that *L. monocytogenes* is more likely to be found in fresh-cut fruits, especially melons. Therefore, food safety surveillance programs are important to guarantee the microbial quality of fresh-cut melons and assure public health maintenance.

As the Centre for Disease Control and Prevention (CDC) recommended, pre-cut fruits should be stored at refrigeration temperatures and for no longer than 10 days, depending on the fruit and only if packaged and stored properly [4,12]. For convenience, since it is difficult to judge whether they are ripe, and also because many specimens are very large, melons are often cut into quarters and halves in the shops, without washing or cleaning, wrapped in plastic cling film and exposed to room temperature. Melon is at particular risk of contamination from pathogens due to its rough peel that enables bacterial attachment and survival, especially if stored at abusive temperatures. The flesh characteristics of melon—near neutral pH (between 5.10 and 6.67), high water activity (between 0.97 and 0.99) and high sugar content [13,14]—also contribute to bacterial activity. Thus, if not handled properly, kept at refrigeration temperatures, or consumed quickly, cut melon may represent a reservoir for pathogenic bacteria and become a potential source of foodborne diseases [3,11].

The objectives of the present work were primarily to study the microbiological quality of *Cucumis melo* L. var. “Piel de Sapo” melons, which were cut and exposed to room temperature in fruit shops in Porto, Portugal. In this country, no information exists about contamination levels of these ready-to-eat products. Secondly, artificial contamination with relevant foodborne pathogens from the peel of “Piel de Sapo” melons was performed to assess whether transfer from the peel to the flesh would be significant during its slicing and after storage at abusive temperatures for 2 days.

## 2. Materials and Methods

### 2.1. Sampling

**Figure 1:**
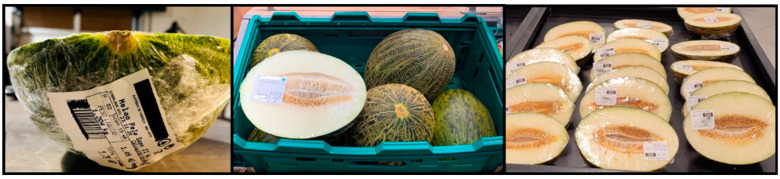
Cut and wrapped melons for sale at the fruit shop.

Twenty-six single-type melons, *Cucumis melo* L. var. “Piel de Sapo”, cut and wrapped in plastic cling film in a shop, were analysed. All samples were exposed to room temperature (with maximum temperatures ranging from 15 to 25 °C) and purchased from September 2020–January 2021. Photos of melons, as purchased, are presented in Figure 1. Samples from eleven local fruit shops (traditional markets, grocery stores or supermarkets) in Porto, Portugal, were assessed. The distribution of the number of samples per shop depended on the availability of the product (from the same species and subjected to the same practice) during the study period. Samples were transported to the laboratory in portable, insulated cold boxes and stored at 4 °C until they were analysed, usually between 8 and 12 h after collection. The plastic cling film and seeds were aseptically removed with a sterilised spoon, and two sample parts were analysed—the flesh of the cut and exposed surface, and the melon peel. Several pieces were taken from each melon sub-part (peel and flesh), and two individual samples composed of randomly selected pieces were prepared for analysis.

### 2.2. Microbial Enumeration and Detection of Pre-Cut Melons

Twenty-five grams of each sample (peel and flesh) were transferred to sterile stomacher bags, in duplicate, and 225 g of half-Fraser broth (Merck, Darmstadt, Germany) or Buffered Peptone Water (BPW, Biokar diagnostics, Beauvais, France) were added to each bag and homogenised in a BagMixer (Interscience, Saint Nom, France) for 1 min. For *Listeria* spp. detection, samples in bags with half-Fraser broth were incubated at 37 °C for 24 h. Samples in bags with BPW were used for microbial enumeration and later incubated at 30 °C for 24 h for *Salmonella* spp. detection.

For microbial enumeration, appropriate decimal dilutions were prepared in Ringer solution (Biokar diagnostics) and plated into the respective culture media. Total viable microorganisms were counted on Plate Count Agar (PCA, Biokar diagnostics) after incubation at 30 °C for 72 h, considering all the colonies grown [15]. Tryptone Bile X-glucuronide (TBX, Biokar diagnostics) agar was used to grow *E. coli* and, after incubation at 44 °C for 24 h, only characteristic blue colonies were counted [16]. Agar Listeria, according to Ottaviani and Agosti (ALOA, bioMérieux, Marcy l’Etoile, France), was used for the enumeration of *L. monocytogenes* and *Listeria* spp. [17], and Baird Parker Agar (BPA, Biokar diagnostics) was used for the growth of staphylococci, and both were incubated at 37 °C for 24 or 48 h [18]. According to the respective ISO standards [17,18], only typical colonies of these two microbial agents must be confirmed. Typical blue-green colonies with or without opaque halo in ALOA for *L. monocytogenes* and *Listeria* spp., respectively, were confirmed according to ISO 11290-1: [17]. Presumptive staphylococci colonies on BPA, characteristic black or grey colonies, shiny and convex surrounded by a light halo with an opalescent ring or non-characteristic black and shiny or grey colonies, with or without a narrow white border where the light area is absent or faintly visible, were confirmed according to ISO 6888-1: [18].

For the detection of *L. monocytogenes* and *Listeria* spp., 0.1 mL of incubated samples in half-Fraser bags were transferred to Fraser tubes (Merck) and incubated at 37 °C for 24 h [19]. To detect *Salmonella* spp., after pre-enrichment in BPW for 24 h, 1 mL was transferred to Muller–Kauffmann Tetrathionate-Novobiocin broth (MKTTN, bioMérieux) and 0.1 mL to Rappaport–Vassiliadis soya peptone broth (RVS, bioMérieux) and incubated for 24 h at 37 °C and 41.5 °C, respectively [20]. Typical colonies were confirmed according to the respective standard protocols.

### 2.3. Identification of Staphylococci by Sanger Sequencing of the 16S rRNA

Four isolates (two from the peel and two from the flesh) of each confirmed colony were identified by 16S rRNA. The DNA was extracted using GRS Genomic DNA Kit Bacteria (Grisp, Porto, Portugal). Polymerase chain amplification of the 16S rRNA gene fragments was performed according to Ferreira da Silva et al. [21] in a T100 Thermal Cycler (BioRad, Algés, Portugal) with 50 μL mixtures using 25 mM MgCl_2_, 10X Taq Buffer with KCl, 100 μM of primers 27F (5′-AGAGTTTGATCCTGGCTCAG-3′) and 1492R (5′-GGTTACCTTGTTACGACTT-3′), 10 mM dNTPs solution, 1 U/μL Taq polymerase and 2 μL of extracted DNA. The following conditions were used: start cycle of 94 °C for 5 min, 30 cycles of denaturation at 94 °C for 30 s, annealing at 55 °C for 30 s and extension at 72 °C for 1.3 min, and a final extension at 72 °C for 1.3 min followed by cooling at 12 °C. DNA previously extracted from a control strain and reactions without template DNA were used as positive and negative controls, respectively. PCR products were purified using a GRS PCR & Gel Purification Kit (Grisp) and used as templates for sequencing. Each purified PCR product was sent to GATC Biotech for DNA sequencing. Sequences obtained were aligned with the sequences in Gene Bank using the BLAST program (http://www.ncbi.nlm.nih.gov, accessed on 25 January 2021) [22].

### 2.4. Effect of Cutting and Storage Temperature of Contaminated Melons on Pathogen Survival

#### 2.4.1. Preparation of Pathogen Inocula and Artificial Contamination of Melons

Cultures of pathogens deposited in the culture collection of the *Escola Superior de Biotecnologia*, Porto, Portugal, were used: seven *L. monocytogenes* strains (2542, FSL N1-227, FSL N3-013, FSL R2-499, FSL J1-031, MF 4077, FSL J1-177), representatives of five *Salmonella* serovars (*Salmonella* Typhimurium 27C, *Salmonella* Enteritidis ESB08, *Salmonella* Seftenberg 775 W, *Salmonella* Infantis M 2106 and *Salmonella* Typhimurium SLM 1) and two *E. coli* strains (*E. coli* ATCC 25922, *E. coli* O157:H7—Vtx negative).

One colony of each pathogen, grown on TSAYE (Biokar Diagnosis) at 37 °C for 24 h, was transferred to TSBYE and incubated overnight at 37 °C. For the final inocula, 1% (*v*/*v*) was taken from the last culture, transferred to fresh TSBYE and incubated at the same conditions. Cocktails with pathogens were prepared by mixing equal volumes of each strain from the same species, measuring the optical density at 600 nm, and adjusting each cocktail to a final level of ca. 10^9^ Colony Forming Unit (CFU)/mL.

Whole melons (*Cucumis melo* L. var. “Piel de Sapo”) were purchased from a fruit shop and transported to the laboratory. For artificial contamination, the entire surface of each melon was wrapped with a gauze previously dipped into each inoculum. Each wrapped melon was stored in sterile stomacher bags at 20 °C for 24 h to allow sufficient contact time for pathogens to transfer and attach from the gauze to the peel (artificial contamination). Taking into account this method of contamination, a high level of each pathogen cocktail (10^9^ CFU/mL) was used to ensure that the initial cell number on the surface of the melon sufficiently exceeded the detection limit of the enumeration technique. Three independent replicates were performed.

#### 2.4.2. Microbiological Analysis of Artificially Contaminated Melons

Each contaminated melon was cut into nine slices using sterile utensils. Three slices were randomly selected to be immediately analysed (day zero) to simulate the cut of the melon and its immediate consumption. The remaining slices were incubated again in new and sterilised stomacher bags at 20 °C and analysed on days 1 and 2 to simulate the consumption of melon previously sliced and left at room temperature (abusive temperature).

For the microbiological analysis of each slice, each 10 g of interior flesh (using randomly selected pieces) was added, individually, to 90 g of BPW and decimal dilutions in Ringer’s solution were performed. Each sample and respective dilutions were spread-plated on Polymyxin Acriflavine Lithium Chloride Ceftazidime Aesculin Mannitol agar (PALCAM, Biokar diagnostics) for the enumeration of *L. monocytogenes*, on RAPID *Salmonella* agar (BioRad) for the enumeration of *Salmonella* spp. and on TBX agar (Biokar diagnostics) for the enumeration of *E. coli*. Each selective culture medium used appropriately for each pathogen cocktail was selected based on current ISO standards or older versions, as in the case of PALCAM for *L. monocytogenes*. All the plates were incubated at 37 °C for 24 or 48 h, and the CFU/g was calculated.

To control microbial loads naturally present in the melon peels, swab samples from the entire surface of each melon were initially taken and immersed in 10 mL Ringer’s solution; 0.1 mL was then spread-plated on each culture media.

### 2.5. Statistical Analysis

Data analysis for microbiological results and enumeration of artificially contaminated melons over time (0, 24, 48 h) were performed using IBM SPSS software (version 28.0). Statistical differences were analysed for significance by one-way analysis of variance (ANOVA) using Tukey’s multiple range tests and independent *t*-tests, respectively. Statistical significance was assumed when *p* < 0.05.

## 3. Results

### 3.1. Microbial Enumeration and Detection of Pre-Cut Melons

Microbiological analysis of the peel and the flesh-cut surface of cut melons was performed; the results are presented in Table 1. For each melon, no significant differences (*p* > 0.05) were found for total viable microorganisms on the flesh and peel of the same sample, despite the significant differences (*p* < 0.05) in melons from different stores. High numbers (>6 log CFU/g) of total viable microorganisms were found. It was observed that for both flesh and peel, eleven samples (1, 8, 9, 10, 11, 12, 13, 15, 20, 23 and 25) presented counts between 6 and 8 log CFU/g and four samples (4, 5, 17 and 21) higher than 8 log CFU/g. Counts higher than 8 log CFU/g were also found in samples 6 and 14, but only in the flesh. In a study performed in Southern Portugal, but with packed cut green melon, Galia melon, cantaloupe melon and watermelon, the authors also found high numbers of aerobic mesophilic microorganisms, ranging from 3.62 to 8.92 log CFU/g [1]. In addition, higher total viable counts were observed on the flesh-cut surfaces than on the peel for 15 samples (Table 1). This scenario was reported by others for cantaloupe and honeydew melons [23,24], where the transfer of microorganisms present on the peel to the flesh can occur when the melon is cut. If fruit processing occurs in the shop without appropriate treatment, packaging and storage, microorganisms might survive and grow exponentially.

*Escherichia coli* was present in four samples (13, 14, 15 and 22), in numbers ranging from 1.00 to 3.85 log CFU/g (Table 1). Two of these samples (14 and 15) were bought from the same store (D). The acceptable microbial range of total *E. coli* in fresh fruits lies between 20 and 100 CFU/g, and *E. coli* O157:H7 must not be detected in 25 g of the sample [25]. Since samples 13 and 15 exceeded this threshold, they were considered unacceptable for human consumption. Additionally, the numbers of *E. coli* found were significantly higher (*p* < 0.05) on the cut surface than on peels, suggesting the possibility of transfer of *E. coli* from peels to the cut surface, and subsequent growth. Other authors also reported this finding for “Western shippers” cantaloupes and *Cucumis melo* L. var “Piel de sapo” [26,27]. Abadias et al. [28] reported populations of 8.9 log CFU/g in fresh-cut melon after one day of storage at 25 °C under air conditions. Ukuku et al. [26] conducted a transfer experiment and found no *E. coli* on the peels of melons, which could somehow indicate that few *E. coli* can be detected on peels but with the potential to thrive on the cut surface after slicing, as was observed with the melon samples analysed in this study.

Despite numbers being below the detection limit for some samples, *Staphylococcus* spp. were also found at levels between 3 and 5 log CFU/g, with several samples (10, 13, 14, 16, 17, 18 and 25) exceeding 5 log CFU/g (Table 1), which is a matter of concern since the production of enterotoxins can occur. Therefore, coagulase-negative *Staphylococcus* spp. should not be ignored but monitored constantly. Most of the isolates found were coagulase-negative staphylococci. 16S rRNA sequencing confirmed that *S. aureus* was present on samples 6, 11, 14, 20, 21 and 24, in numbers between 1.80 and 3.52 log CFU/g (Table 1). For a fresh fruit to be considered satisfactory or acceptable, the presence of *S. aureus* should be lower than 20 CFU/g or between 20 and 100 CFU/g, respectively [25]. Hence, four samples (6, 14, 20 and 24) were considered unsatisfactory, and sample 21 was at the edge of acceptance (1.80 log CFU/g). As far as we know, few studies are available in the literature about the detection of *S. aureus* in these products. However, Park et al. [29] reported the absence of *S. aureus* in 123 whole-melon sample peels in Korea. Considering the results obtained in this study, the potential occurrence of melons contaminated with *S. aureus* should be monitored. While the small number of staphylococci in food is not a direct health hazard, its presence in these pre-cut products left at room temperature in fruit shops can quickly become a problem when taken home and kept at abusive temperatures by consumers.

No *Salmonella* spp. or *Listeria* spp. were detected in the samples which were all considered satisfactory for these microbial parameters [25]. Regarding *Salmonella*, other authors reported their absence on pre-cut green melon, Galia melon, cantaloupe, honeydew and watermelon [1,11]. However, unlike the present study, their samples were packed. Regarding *L. monocytogenes*, a quantitative limit of 100 CFU/g is set in Regulation 1441/2007 [30] for criterion 1.3 (RTE foods not able to support the growth of *L. monocytogenes*) and for criterion 1.2 (RTE foods able to support the growth of *L. monocytogenes*) when the manufacturer is able to demonstrate, to the satisfaction of the competent authority, that their product will not exceed the 100 CFU/g limit throughout its shelf-life. Even though it was not found in this study, and in agreement with other studies on cantaloupe and melon [29,31], the potential growth of *L. monocytogenes* on cut melons and its correlation with temperature was already reported for “Rocky Ford” cantaloupes [32].

### 3.2. Identification of Staphylococci by Sanger Sequencing of 16S rRNA

The results for 16S rRNA sequencing of *Staphylococcus* spp. are presented in Table S1. The most prevalent species was *Staphylococcus xylosus*, with an occurrence of 21% on peels and 21% on the cut surface, followed by *S. aureus*, with an occurrence of 5% on peels and 4% on the cut surface. For the remaining isolates, 14% were confirmed as belonging to other genera; *Bacillus* spp. were the most prevalent, followed by *Microbacterium arborescens*, *Cellulosimicrobium funkei*, *Isoptericola cucumis*, *Lysinibacillus macrolides* and *Aerococcus urinaeequi*.

Despite being the second major group found, the occurrence of *S. aureus* was less when compared with the growth observed directly from BPA plates. One hypothesis is the ambiguity in distinguishing between the opaque zone produced by lecithinase and the clear zone produced by lipase shown on yellowish agar. It is, therefore, possible that the lipase production *Staphylococcus* spp., such as *S. epidermidis*, *S. warneri* and *S. xylosus* that appeared in the sequencing results, were mistaken as typical colonial traits of *S. aureus* [33]. This assumption, in turn, reinforces the importance of other methods of confirmation (e.g., 16S rRNA sequencing), which is not considered in ISO standards for the enumeration of *S. aureus*.

*Staphylococcus xylosus*, the most abundant *Staphylococcus* spp. found in all the samples, is commonly used in the production of fermented sausages to confer colour and aroma [34]. They are a type of commensal bacteria that can be found on the skin or mucous of mammals or birds [35] and, despite being generally categorised as non-pathogenic, certain strains of *S. xylosus* might be related to human infections [36]. When comparing the variety of species found in the peel or cut surface of melons, it was observed that almost all species of *Staphylococcus* spp. were found equally distributed, with *S. edaphicus* and *S. epidermidis* isolated only on the cut surface.

*Bacillus* spp. were also found (Table S1), some of which are common foodborne pathogens [37]. The species *Bacillus nealsonii* was newly isolated during spacecraft assembling in 2003 [38], while *Bacillus circulans* was known as a human pathogen and fatal for immunocompromised patients [39]. Other confirmed species were *Bacillus cereus*, a foodborne pathogen that can produce toxins causing vomiting (emetic syndrome) or diarrhoea (diarrhoeal syndrome) [40], and *Bacillus zhangzhouensis* first isolated from aquaculture in 2016 [41] and reported to have the ability to produce keratinase and aid in the degradation of feathers from poultry industry waste [42]. Similar to *Staphylococcus* spp., certain species of *Bacillus* spp. have the ability to reduce tellurite. This is one of the compounds that makes BPA selective and differential for *Staphylococcus* spp [43]. For this reason, if proper confirmatory tests are not performed, some *Bacillus* spp. may be misidentified as *Staphylococcus* spp. Regarding the other Gram-positive species found, *Microbacterium arborescens* has been under studied, but other species within the same genus were reported to be yellow-pigmented [44] and considered to be pathogenic for humans, causing endophthalmitis [44,45]. *Cellulosimicrobium funkei* was proposed as a new species in 2006 [46] and reported as an opportunistic pathogen in humans [47]. First isolated in 2016 from the root of cucumber, *Isoptericola cucumis* remained little studied [48]. *Lysinibacillus macrolides* were studied for its capability to degrade chloro-benzoic acids (CBAs), a group of toxic chemicals released in the use of pesticides or herbicides, as well as polychlorinated biphenyls (PCBs) [49]. Originally named *Pediococcus urinaeequi*, this Gram-positive bacterium was reclassified in 2005 as *Aerococcus urinaeequi* due to traits shared with *Aerococcus* spp. [50]. Bacteria of this genus are believed to be human pathogens, causing urinary tract infection and inflammation of the endocardium [51].

Among the various bacterial species isolated from pre-cut melons, some are rarely found in these products, suggesting cross-contamination [27]. For example, *S. epidermidis* was considered a commensal species of the human skin [52]; *B. cereus*, commonly found in other products, were linked to food poisoning due to ingestion of meat, dairy, rice or pasta products [53].

When comparing the variety of species found in the peel or cut surface of melons, it was observed that almost all species of *Staphylococcus* spp. were found equally distributed, with only *S. edaphicus* and *S. epidermidis* isolated only on the cut surface. Regarding other microorganisms, a greater variety was observed on the cut surface, which may be due to their contact with the environment and the probable ability of these species to be transferred to the interior of the melons.

### 3.3. Effect of Cutting and Storage Temperature of Contaminated Melons on Pathogens Survival

Melon peels were contaminated with cocktails of *L. monocytogenes*, *E. coli* and *Salmonella*. An experiment was conducted to simulate the cutting of contaminated melons in-store or in consumer homes and to determine the potential passage of each foodborne pathogen to the melon flesh and subsequent growth.

**Figure 2:**
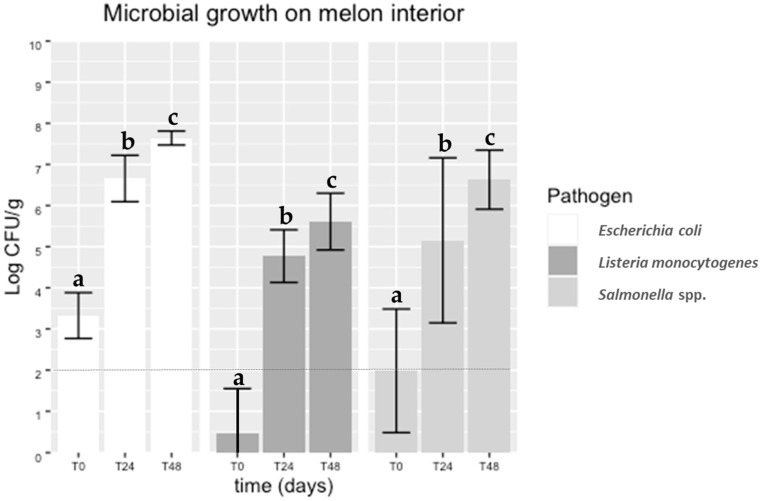
Microbial growth of *Salmonella* spp., *Listeria monocytogenes* and *E. coli* cocktails on the flesh of melons after 0, 24 and 48 h of cutting. The detection limit of the enumeration technique was 2 log CFU/g (dotted line). Equivalent lowercase letters mean no significant differences (*p* > 0.05).

Microbial loads found for each pathogen in the interior of artificially contaminated melons immediately after cutting (0 h) and during storage (after 24 and 48 h) at 20 °C are reported in Figure 2. It was observed that the pathogens were transferred from the contaminated peel to the interior of the melons, a finding supported by literature on netted melons [54]. Over the storage time, significant differences were observed (*p* < 0.05) between the counts obtained from slices immediately cut (time 0 h) and after 24 and 48 h at 20 °C, for each pathogen. At time 0 h, the pathogens were recovered in numbers lower than those in the gauze used for artificial surface contamination of the melons. Other authors used an immersion method for melon inoculation in their studies [14,26]. Nevertheless, after 24 h stored at 20 °C, an increase of about 4 log CFU/g was observed in all the pathogens.

Even though different initial cell recoveries (about 1 log CFU/g difference) were observed between both pathogens (Figure 2), the increase in *Salmonella* spp. and *E. coli* on the melon flesh was similar during storage time. Considering the infectious dose of *Salmonella* spp. (from 10^3^ to 10^5^ CFU depending on serovar) [55] and the low infectious doses of *E. coli* O157:H7 (less than 100 cells), these results are a cause for concern. Several studies regarding the contamination of melons with *Salmonella* spp. have been performed. Ukuku and Sapers [24] reported an increasing *Salmonella* spp. growth from 2.0 to 3.6 log CFU/g on “Western shippers” melon pieces stored at 10 °C and 22 °C (abusive temperature), respectively. Furthermore, increases of 1.5 log CFU/g (fresh-cut honeydew) and 3 log CFU/g (fresh-cut cantaloupe) were observed in samples stored for 12 days at 10 °C and 22 °C, respectively.

Counts of *L. monocytogenes* (Figure 2) immediately after cutting (0 h) were above the detection limit of the enumeration technique. However, an almost 4 log CFU/g increase was observed during storage, with counts exceeding 5 log CFU/g. This finding was also consistent with a recent study on cantaloupes reporting a 4 log CFU/g increase in *L. monocytogenes*, from 5 log CFU/g to 9 log CFU/g after storage for 3 days at 25 °C [56]. On the other hand, Nyarko et al. [32] reported an increase of 1.0 and 3.0 log CFU in *L. monocytogenes* per “Rocky Ford” melon cube after seven days of storage at 4 °C and 10 °C, respectively. Taking into account that 92% of invasive listeriosis resulted from ingestion of more than 2000 CFU/g of ready-to-eat food [57], it is essential to store cut melons at an appropriate storage temperature to guarantee that the number of these foodborne pathogens, if present, will not reach sufficient levels to cause illness.

## 4. Conclusions

The microbial quality of the cut melons assessed in the Porto Metropolitan Area was shown to be less than ideal, with one sample being classified as unacceptable for consumption and four as unsatisfactory, out of the 26 samples in total used in this study. Even though *Salmonella* spp. and *L. monocytogenes* were not detected in any samples, *E. coli* and staphylococci were isolated from several samples, at levels above the established limits for microbiological quality. Additionally, the artificial contamination of melons in this preliminary study also demonstrated that foodborne pathogens, such as *Salmonella* spp., *E. coli* and *L. monocytogenes*, can be transferred from the peel to the interior of the fruit. Although the transfer rate seems to be low, pathogen levels can increase to about 4 log CFU/g if the fruit is not consumed immediately and stored in abusive temperatures. Therefore, effective practices to prevent contamination, cross-contamination and bacterial growth on cut fresh melon must be adopted. At the retail level, melons should be cut in small batches and kept refrigerated for short periods.

## Figures and Tables

**Table 1:** Microbiological analysis (log CFU/g) of surface and peel of cut melons.

Sample	Store	Area	Total Microorganisms	*E. coli*	Coagulase-Negative Staphylococci	Coagulase-Positive Staphylococci
1	A	flesh	6.04 ± 0.06 abcA	<1.00 ± 0.00 aA	3.00 ± 0.10 cA	2.45 ± 0.31 bA
		peel	7.03 ± 0.09 abcA	<1.00 ± 0.00 aA	4.05 ± 0.20 cA	3.84 ± 0.46 bA
2	A	flesh	4.69 ± 0.30 abcA	<1.00 ± 0.00 aA	>3.72 ± 0.65 cA	<1.00 ± 0.00 aA
		peel	4.74 ± 1.04 abcA	<1.00 ± 0.00 aA	4.19 ± 0.16 cA	1.00 ^E^ ± 1.41 aA
3	A	flesh	3.62 ± 0.87 abcA	<1.00 ± 0.00 aA	0.95 ^E^ ± 1.35 cA	<1.00 ± 0.00 aA
		peel	3.77 ± 0.47 abcA	<1.00 ± 0.00 aA	1.00 ^E^ ± 1.41 cA	<1.00 ± 0.00 aA
4	A	flesh	>8.48 ± 0.00 abcA	<1.00 ± 0.00 aA	3.96 ± 0.05 cA	0.50 ^E^ ± 0.71 aA
		peel	8.47 ± 0.16 abcA	<1.00 ± 0.00 aA	3.69 ± 0.55 cA	1.24 ± 0.34 aA
5	A	flesh	8.55 ± 0.09 abcA	<1.00 ± 0.00 aA	3.08 ± 0.05 cA	1.00 ^E^ ± 1.41 aA
		peel	>8.48 ± 0.00 abcA	<1.00 ± 0.00 aA	4.13 ± 1.02 cA	2.90 ± 0.85 bA
6	A	flesh	8.72 ± 0.39 abcA	<1.00 ± 0.00 aA	4.83 ± 0.21 cA	3.59 ± 0.14 bA
		peel	7.65 ± 0.11 abcA	<1.00 ± 0.00 aA	4.18 ± 0.05 cA	2.82 ± 0.20 * bA
7	B	flesh	4.76 ± 0.71 abcA	<1.00 ± 0.00 aA	4.10 ± 0.15 bcA	2.30 ± 0.00 bA
		peel	5.17 ± 0.98 abcA	<1.00 ± 0.00 aA	4.02 ± 0.03 bcA	3.12 ± 0.23 bA
8	B	flesh	7.26 ± 0.26 abcA	<1.00 ± 0.00 aA	3.29 ± 0.46 bcA	<1.00 ± 0.00 aA
		peel	6.86 ± 0.91 abcA	<1.00 ± 0.00 aA	3.38 ± 0.24 bcA	<1.00 ± 0.00 aA
9	B	flesh	7.69 ± 0.18 abcA	<1.00 ± 0.00 aA	3.28 ± 0.18 bcA	<1.00 ± 0.00 aA
		peel	6.45 ± 0.21 abcA	<1.00 ± 0.00 aA	3.12 ± 0.18 bcA	0.65 ^E^ ± 0.92 aA
10	C	flesh	6.98 ± 0.63 abcA	<1.00 ± 0.00 aA	>4.94 ± 0.35 abA	<1.00 ± 0.00 aA
		peel	7.39 ± 0.20 abcA	<1.00 ± 0.00 aA	>5.05 ± 0.18 abA	3.50 ± 0.28 aA
11	C	flesh	7.41 ± 0.63 abcA	<1.00 ± 0.00 aA	3.57 ± 0.54 abA	<1.00 ± 0.00 aA
		peel	5.82 ± 0.04 abcA	<1.00 ± 0.00 aA	3.26 ± 0.21 * abA	<1.00 ± 0.00 aA
12	C	flesh	6.89 ± 0.68 abcA	<1.00 ± 0.00 aA	4.85 ± 0.03 abA	2.80 ± 1.13 aA
		peel	6.45 ± 0.36 abcA	<1.00 ± 0.00 aA	4.20 ± 0.11 abA	>3.09 ± 0.13 aA
13	C	flesh	6.87 ± 0.32 abcA	2.13 ± 1.59 bB	>5.18 ± 0.00 abA	>4.82 ± 0.51 aA
		peel	6.45 ± 0.17 abcA	1.43 ± 0.60 bA	>5.18 ± 0.00 abA	>4.59 ± 0.83 aA
14	D	flesh	8.77 ± 0.06 abcA	1.39 ± 0.55 bA	>5.18 ± 0.00 abcA	3.52 ± 0.31 * bA
		peel	6.95 ± 0.06 abcA	<1.00 ± 0.00 aA	4.17 ± 0.24 abcA	2.75 ± 0.16 * bA
15	D	flesh	6.70 ± 0.23 abcA	3.85 ± 0.78 cB	4.09 ± 0.4 abcA	1.15 ± 0.21 aA
		peel	6.60 ± 0.03 abcA	2.45 ± 2.05 bC	3.97 ± 0.13 abcA	2.01 ± 0.75 aA
16	E	flesh	5.44 ± 0.46 abcA	<1.00 ± 0.00 aA	>5.24 ± 0.08 aA	2.30 ± 0.99 aA
		peel	6.34 ± 0.03 abcA	<1.00 ± 0.00 aA	>5.15 ± 0.04 aA	1.74 ± 2.46 aA
17	E	flesh	>8.48 ± 0.00 abcA	<1.00 ± 0.00 aA	>5.18 ± 0.00 aA	<1.00 ± 0.00 aA
		peel	8.06 ± 0.27 abcA	<1.00 ± 0.00 aA	>5.18 ± 0.00 aA	<1.00 ± 0.00 aA
18	E	flesh	5.88 ± 0.07 abcA	<1.00 ± 0.00 aA	5.31 ± 0.19 aA	2.21 ± 3.12 aA
		peel	7.22 ± 1.56 abcA	<1.00 ± 0.00 aA	5.61 ± 0.30 aA	<1.00 ± 0.00 aA
19	E	flesh	5.21 ± 0.07 abcA	<1.00 ± 0.00 aA	5.81 ± 0.11 aA	<1.00 ± 0.00 aA
		peel	5.17 ± 0.09 abcA	<1.00 ± 0.00 aA	5.40 ± 0.27 aA	<1.00 ± 0.00 aA
20	F	flesh	7.95 ± 0.07 aA	<1.00 ± 0.00 aA	3.60 ± 0.08 bcA	2.54 ± 0.76 * aA
		peel	7.46 ± 0.16 aA	<1.00 ± 0.00 aA	4.04 ± 0.23 bcA	2.96 ± 0.06 aA
21	F	flesh	8.83 ± 0.20 aA	<1.00 ± 0.00 aA	4.13 ± 0.98 bcA	3.07 ± 0.10 * bA
		peel	8.92 ± 0.15 aA	<1.00 ± 0.00 aA	4.08 ± 0.23 bcA	1.80 ± 0.28 * aB
22	G	flesh	4.58 ± 0.07 bcA	1.00 ^E^ ± 0.00 aA	3.01 ± 0.38 bcA	>1.59 ± 2.25 aA
		peel	5.42 ± 0.06 bcA	<1.00 ± 0.00 aA	>4.15 ± 0.04 bcA	<1.00 ± 0.00 aA
23	H	flesh	7.56 ± 0.15 abA	<1.00 ± 0.00 aA	3.25 ± 0.84 bcA	1.09 ± 1.54 aA
		peel	>8.22 ± 0.36 abA	<1.00 ± 0.00 aA	3.55 ± 0.84 bcA	>2.63 ± 0.78 aA
24	I	flesh	5.41 ± 0.77 bcA	<1.00 ± 0.00 aA	3.86 ± 0.65 abcA	>1.59 ± 2.25 * aA
		peel	5.40 ± 0.46 bcA	<1.00 ± 0.00 aA	4.30 ± 0.86 abcA	>3.18 ± 0.00 aB
25	J	flesh	6.69 ± 0.34 abcA	<1.00 ± 0.00 aA	5.75 ± 0.53 aA	<1.00 ± 0.00 aA
		peel	6.57 ± 0.04 abcA	<1.00 ± 0.00 aA	>5.94 ± 0.33 aA	<1.00 ± 0.00 aA
26	K	flesh	4.95 ± 0.72 cA	<1.00 ± 0.00 aA	3.87 ± 0.01 abcA	<1.00 ± 0.00 aA
		peel	4.42 ± 0.35 cA	<1.00 ± 0.00 aA	4.15 ± 0.60 abcA	<1.00 ± 0.00 aA

Legend: Mean ± standard deviation. Equivalent capital letters, by column, mean no significant differences between flesh and peel for the same sample (*p* > 0.05); equivalent lowercase letters, by column, mean no significant differences between stores (*p* > 0.05). Store A—traditional market, Stores B to J—grocery stores, Store K—hypermarket. * Presence of *S. aureus*; ^E^ Estimated number for low numbers [16,18]; no *Listeria* spp. and *Salmonella* were detected; no significant differences were found between samples collected in different months (*p* > 0.05).

## Data Availability

Data is contained within the article.

## Supplementary Materials

The following supporting information can be downloaded at: https://www.mdpi.com/article/10.3390/foods11244010/s1, Table S1: 16S rRNA sequencing results of possible *Staphylococcus* spp.
