# Supplementary material for: Microbiological Safety of Cut Melons Sold in Portuguese Retail Markets: A Pilot Study
Source: Foods. 2022 Dec 11;11(24):4010. doi: 10.3390/foods11244010 (PMC9777610; doi:10.3390/foods11244010)
Supplement: Supplementary file 1 [file foods-11-04010-s001.zip › foods-1977257-supplementary.pdf]

Table S1. 16S rRNA sequencing results of possible *Staphylococcus* spp.

Sample		Species	Similarity (%)
1	flesh	*Staphylococcus xylosus*	99.5
		Staphylococcus xylosus	99.7
	peel	*Staphylococcus xylosus*	99.8
		Staphylococcus xylosus	99.6
2	flesh	*Microbacterium arborescens*	99.8
		Cellulosimicrobium funkei	99.2
	peel	*Isoptericola cucumis*	96.7
		Microbacterium arborescens	99.8
4	flesh	*Staphylococcus xylosus*	99.9
		Staphylococcus edaphicus	98.9
	peel	*Staphylococcus xylosus*	99.7
5	flesh	*Staphylococcus succinus*	99.8
		Staphylococcus xylosus	99.9
	peel	*Staphylococcus xylosus*	100.0
		Staphylococcus xylosus	100.0
6	flesh	*Staphylococcus warneri*	100.0
	peel	*Staphylococcus warneri*	100.0
		Staphylococcus aureus	100.0
7	flesh	*Bacillus nealsonii*	98.8
		Bacillus circulans	98.8
	peel	*Bacillus nealsonii*	98.2
		Bacillus nealsonii	98.9
8	flesh	*Aerococcus urinaeequi*	99.9
		Staphylococcus succinus	99.9
	peel	*Staphylococcus equorium*	100.0
9	flesh	*Staphylococcus pasteuri*	100.0
		Staphylococcus xylosus	99.9
	peel	*Staphylococcus pasteuri*	100.0
		Staphylococcus pasteuri	100.0
10	flesh	*Lysinibacillus macroides*	99.4
		Bacillus cereus	99.8
	peel	*Bacillus cereus*	99.6
		Bacillus cereus	99.1
11	flesh	*Staphylococcus edaphicus*	99.9
		Staphylococcus epidermidis	100.0
	peel	*Staphylococcus aureus*	99.9
		Staphylococcus aureus	99.9
12	flesh	*Staphylococcus xylosus*	99.8
		Staphylococcus xylosus	100.0
	peel	*Staphylococcus xylosus*	99.7
		Staphylococcus xylosus	99.9
13	flesh	*Staphylococcus xylosus*	100.0
		Staphylococcus xylosus	100.0
	peel	*Staphylococcus xylosus*	99.8
		Staphylococcus xylosus	100.0
14	flesh	*Staphylococcus xylosus*	99.9
		Staphylococcus aureus	99.7
	peel	*Staphylococcus xylosus*	99.0

		Staphylococcus aureus	99.5
15	flesh	*Staphylococcus xylosus*	99.8
		Staphylococcus xylosus	98.1
	peel	*Staphylococcus xylosus*	100.0
		Staphylococcus xylosus	99.6
16	flesh	*Staphylococcus xylosus*	99.7
		Staphylococcus xylosus	100.0
	peel	*Staphylococcus xylosus*	99.9
17	flesh	*Staphylococcus saprophyticus*	99.5
	peel	*Staphylococcus saprophyticus*	99.9
		Staphylococcus xylosus	99.9
18	flesh	*Staphylococcus xylosus*	100.0
		Staphylococcus xylosus	100.0
	peel	*Staphylococcus xylosus*	100.0
19	flesh	*Staphylococcus sciuri*	99.8
		Staphylococcus sciuri	99.6
	peel	*Staphylococcus sciuri*	99.9
		Staphylococcus sciuri	99.8
20	flesh	*Staphylococcus aureus*	99.9
	peel	*Staphylococcus xylosus*	100.0
		Staphylococcus xylosus	100.0
21	flesh	*Staphylococcus aureus*	99.6
		Staphylococcus xylosus	99.1
	peel	*Staphylococcus aureus*	99.2
22	flesh	*Staphylococcus saprophyticus*	99.8
		Staphylococcus xylosus	100.0
	peel	*Staphylococcus saprophyticus*	99.3
		Staphylococcus xylosus	100.0
23	flesh	*Staphylococcus warneri*	99.8
		Staphylococcus xylosus	99.9
	peel	*Staphylococcus xylosus*	100.0
		Bacillus zhangzhouensis	99.7
24	flesh	*Staphylococcus xylosus*	99.4
		Staphylococcus aureus	99.4
	peel	*Staphylococcus sciuri*	100.0
		Staphylococcus xylosus	99.6
25	flesh	*Staphylococcus xylosus*	99.9
	peel	*Staphylococcus xylosus*	100.0
		Staphylococcus succinus	100.0
26	flesh	*Staphylococcus equorium*	100.0
	peel	*Staphylococcus equorium*	100.0
		Staphylococcus equorium	100.0
